# Inter-organelle membrane contact sites: implications for lipid metabolism

**DOI:** 10.1186/s13062-020-00279-y

**Published:** 2020-11-11

**Authors:** Jean E. Vance

**Affiliations:** Department of Medicine and Group on Molecular and Cell Biology of Lipids, University of Alberta, Edmonton, AB T6G 2S2 Canada

**Keywords:** Membrane contact sites, Phospholipid transport, Cholesterol transport, Mitochondria, Mitochondria-associated membranes (MAM), Endoplasmic reticulum, Plasma membrane

## Abstract

This article supplements a recent Perspective by Scorrano et al. in *Nature Communications* [10 [ (1)]:1287] in which the properties and functions of inter-organelle membrane contact sites were summarized. It is now clear that inter-organelle membrane contact sites are widespread in eukaryotic cells and that diverse pairs of organelles can be linked via unique protein tethers. An appropriate definition of what constitutes an inter-organelle membrane contact site was proposed in the Perspective. In addition, the various experimental approaches that are frequently used to study these organelle associations, as well as the advantages and disadvantages of each of these methods, were considered. The nature of the tethers that link the pairs of organelles at the contact sites was discussed in detail and some biological functions that have been ascribed to specific membrane contact sites were highlighted. Nevertheless, the functions of most types of organelle contact sites remain unclear. In the current article I have considered some of the points raised in the Perspective but have omitted detailed information on the roles of membrane contact sites in biological functions such as apoptosis, autophagy, calcium homeostasis and mitochondrial fusion. Instead, I have provided some background on the initial discovery of mitochondria-endoplasmic reticulum membrane contact sites, and have focussed on the known roles of membrane contact sites in inter-organelle lipid transport. In addition, potential roles for membrane contact sites in human diseases are briefly discussed.

## Discussion

### Background

The Perspective on membrane contact sites that was recently published in *Nature Communications* [1] summarizes many of the recent advances in our understanding of inter-organelle membrane contact sites. It is now apparent that membrane contact sites have distinct protein, and probably lipid [2], compositions and are widespread in eukaryotic cells from yeast to plants to mammals [reviewed in [3–5]]. Many types of organelles in eukaryotic cells form heterotypic membrane contact sites that have been described and visualized in cells over several decades. For example, contact sites have been observed that link the endoplasmic reticulum (ER) with other organelles such as mitochondria, Golgi, plasma membranes, endosomes, lipid droplets and peroxisomes. Usually, the inter-organelle association is transient and dynamic but can sometimes be stable. The spacing between the two organelles appears to generally be in the range of 10 to 80 nm. It is likely, however, that a specific distance between the two organelles does not necessarily imply the existence, or lack of existence, of a membrane contact site. Importantly, the association between two organelle membranes at the contact site is not the result of a membrane fusion event, although fusion of membranes can occur when two membranes approach within a distance of 1–2 nm. An interesting possibility that was raised in the Perspective, and one that deserves further consideration, is that a contact site between two membraneous organelles might contain a factor that inhibits the fusion between the two organelle membranes.

The organelles that form membrane contact sites are linked via protein tethers composed of multiple classes of proteins. Many of the proteins in the tethers of different types of membrane contact sites have similar properties and/or functions. For example, tethers can include structural proteins (some of which contain motifs that can bridge the two membranes), functional proteins (such as ion channels and lipid transfer proteins), as well as putative regulatory proteins. Several proteomic analyses of the proteins in the membrane tethers that link the ER with mitochondria have been reported [6–8]. However, neither a proteomic, nor a lipidomic, profile has yet been reported for the tethers that link other organelle pairs. Thus, important future research goals include identification and comparison of the protein compositions of the different types of inter-organelle tethers, as well as their biological functions.

An analysis of factors that regulate the formation and dissolution of membrane contact sites is another key area for future research. As a step in this direction, short artificial inter-membrane protein tethers were constructed and expressed in yeast, thereby increasing the formation of ER-mitochondria contact sites. Phospholipid transport into mitochondria from the ER (an established function of these contact sites: see Section 3) was correspondingly increased [9]. Interestingly, the loss of a single protein component of a tether does not necessarily abolish the association between the two organelles. For example, in one case, the contact sites between the ER and mitochondria were disrupted only when six different types of tethering proteins in the contact sites were simultaneously eliminated [10]. However, metabolic changes can alter the number of contact sites in a cell, as well as the spacing between the organelles [11].

The Perspective [1] also provided a useful practical listing of the features that define an inter-organelle membrane contact site. The common characteristics of contact sites were proposed to be: (i) close juxtaposition between the two membrane-bound organelles (or monolayer-bound lipid droplets) with a separation of 10 to 80 nm between the organelles; (ii) lack of fusion between the two membranes at the contact site; (iii) tethering of the two organelles via distinct sets of proteins (and probably lipids); (iv) assignment of a specific biological function to the contact site. Clearly, these criteria are important but not all of these properties have yet been established for all types of contact sites that have been described.

Other important practical information provided by the Perspective [1] is a detailed discussion of the advantages and disadvantages of the various techniques commonly used for studying membrane contact sites. It is noteworthy that membrane contacts often occur only transiently and can be much smaller than the optical resolution limit of the microscope, thereby potentially limiting the use of this technique. Consequently, a combination of several complementary techniques, such as fluorescence microscopy and the gold-standard, transmission electron microscopy, as well as subcellular fractionation [12] and biochemical analyses, is highly recommended for studying membrane contact sites. The use of quantitative electron microscopy morphometric analysis is particularly useful for in vivo evaluation of inter-organelle contacts.

### Early studies on membrane contact sites

Electron microscopy data from the 1950s [13–15] and early subcellular fractionation studies [16–19] led to the proposal that inter-organelle membrane contact sites are abundant in living cells and might be biologically functional. Nevertheless, skepticism and vigorous debate ensued and these findings were generally overlooked for many years. However, subsequent studies have provided more tangible and convincing evidence that inter-organelle membrane contacts are, indeed, common features of eukaryotic cells from yeast [20, 21] to plants [4] to mammals [reviewed in [5]]. It is now evident that membrane contacts can provide a platform for diverse biological functions including regulation of calcium homeostasis [22, 23] and inter-organelle lipid transport [19, 24–26]. Thus, a gradual scientific evolution occurred over a 70-year time-span, during which the idea that membrane contact sites do exist in cells and are required for biological functions, became generally accepted. This re-evaluation of data is an example of how initial challenges to interpretation of scientific data can become modified over many years, leading eventually to an updated formulation of a valid model.

**Fig. 1:**
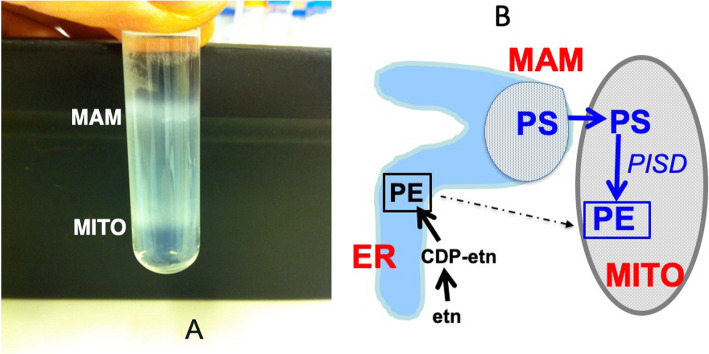
Isolation of MAM and its role in phospholipid import into mitochondria. **a** Rat liver was homogenized and mitochondria (MITO, lower band) were isolated by Percoll gradient ultracentrifugation [19]. The upper band shown on the gel contains the MAM, a specific domain of the ER that has high affinity for mitochondria. **b** Biosynthesis of phosphatidylserine (PS) and PS import into mitochondria. PS is made via synthase-1 and -2 primarily in the MAM. Newly-made PS is exported from MAM to mitochondria (MITO) and converted therein to phosphatidylethanolamine (PE) via the mitochondrial inner membrane enzyme, PS decarboxylase (*PISD*). PE is also made in the ER from ethanolamine (Etn) but this source of PE is not readily imported into mitochondria (dotted line)

In the 1950s, electron microscopy data from rat liver revealed a close juxtaposition between the ER and mitochondria [13–15]. However, at that time no biological function was proposed for these associations. Subcellular fractionations of rat liver, using standard centrifugation methods, had routinely resulted in the isolation of mitochondria that were “contaminated” with ER membranes. However, in the 1990s, a method for a more rigorous purification of rat liver mitochondria was developed that involved Percoll gradient centrifugation [27]. This method yielded purified mitochondria that were devoid of most of the contaminating ER. However, the ER “contaminant” was also collected from the Percoll gradient as a separate entity (Fig. 1a), instead of being discarded; this ER fraction was named “mitochondria-associated membranes” or MAM [19, 28, 29]. The isolated MAM possessed many, but not all, properties that are typical of the ER [19, 26, 29, 30]. For example, the protein composition of MAM was similar, but not identical, to that of the bulk of ER and was quite distinct from that of mitochondria. These data indicated that the MAM was a specific domain of the ER. Moreover, these fractionation studies revealed that the close association between the MAM sub-fraction of the ER and mitochondria was not due to a membrane fusion event but represented a reversible physical association that linked an element of the ER to mitochondrial outer membranes. Later studies showed that these ER-mitochondria contact sites are abundant in cells and that 5 to 20% of mitochondria lie in close proximity to the ER [23].

### Role of membrane contacts in lipid metabolism

The isolation of MAM led to the tantalizing idea that a close juxtaposition of elements of the ER with mitochondria might be required for specific biological functions, such as the inter-organelle (i.e. ER to mitochondria) transport of lipids. The majority of membrane lipid synthesis occurs on ER membranes whereas mitochondria per se have only a limited capacity for lipid synthesis and must, therefore, import most of their lipids from the ER. Notable exceptions are a specific pool of phosphatidylserine (PS)-derived phosphatidylethanolamine (PE), and cardiolipin, both which are synthesized in mitochondrial inner membranes. The mechanisms by which hydrophobic lipid molecules are transported between organelles through the aqueous cytosol were at that time, and still are, not well understood. Potential mechanisms for transport of lipids from their sites of synthesis in the ER to other organelles (such as mitochondria and the plasma membrane) include free diffusion through the cytosol, transport via vesicles, transport via specific cytosolic lipid transfer proteins, and close apposition or fusion between the two organelles. However, since the inter-organelle diffusion of hydrophobic lipids through the aqueous cytosol is energetically unfavourable, free diffusion is an unlikely method for inter-organelle transport of most lipids. Moreover, genetic elimination of several abundant cytosolic lipid transport proteins that can transfer lipids between membranes in vitro, did not significantly alter membrane lipid composition of cells or tissues. In addition, vesicle transport of proteins with their associated lipids is not known to be a major delivery process to mitochondria [reviewed in [31]]. We, therefore, tested the hypothesis that the physical juxtaposition between mitochondrial outer membranes and the ER (MAM) mediates the import of lipid molecules from the ER into mitochondria.

Phosphatidylethanolamine (PE) is a highly abundant mitochondrial phospholipid that is essential for normal mitochondrial functions [32, 33]. The majority of PE in eukaryotic cells is produced by two distinct biosynthetic pathways (Fig. 1b): (i) the CDP-ethanolamine pathway, the final step of which occurs on ER membranes [reviewed in [34]], and (ii) a pathway by which phosphatidylserine (PS) is decarboxylated to PE in mitochondrial inner membranes [reviewed in [34]]. Interestingly, a large majority of the PE that resides in mitochondrial membranes is made in situ in mitochondrial inner membranes via the decarboxylation of PS that is made in ER membranes. In contrast, PE that is generated in the ER from CDP-ethanolamine (Fig. 1b) is largely excluded from being imported into mitochondria [26, 35]. Another lipid that is produced in the ER and imported into mitochondria is phosphatidic acid which can be converted to cardiolipin in mitochondria. However, the mechanism by which phosphatidic acid is transported from the ER to mitochondria has not yet been established.

As an approach to understanding the mechanism by which phospholipids such as PS are imported into mitochondria, we performed a series of experiments in permeabilized cells and in in vitro reconstituted systems of organelles. These studies utilized an assay that quantifies the mitochondrial import of PS from its site of synthesis in the ER and the subsequent decarboxylation of PS to PE [29] (Fig. 1b). Experiments in mutant fibroblasts and other mammalian cells showed that the two enzymes that synthesize PS (PS synthase-1 and PS synthase-2) are highly enriched in MAM compared to the bulk of the ER [36]. The PS transport experiments revealed that newly-synthesized PS, rather than pre-existing PS, is preferentially channeled into mitochondria for conversion to PE [26, 29]. Importantly, when PS decarboxylation in mitochondria was inhibited, newly-made PS accumulated in the MAM [26, 37, 38]. In addition, genetic elimination of the PS decarboxylation pathway in mice caused embryonic lethality [32]. Furthermore, attenuation of PS decarboxylase activity in mammalian cells reduced the PE content of mitochondria and drastically altered mitochondrial morphology and activity, despite the active synthesis of PE in the ER by the alternative CDP-ethanolamine pathway [33]. These data established that PS import into mitochondria via MAM is essential for mouse viability and mitochondrial functions. The experiments also demonstrated that the pool of PE synthesized from PS in mitochondria is not functionally equivalent to the pool of PE that is made in the ER. Similar experiments in yeast also revealed a crucial role for PS import into mitochondria and membrane contact between the ER and mitochondria for optimal functioning of mitochondria [20, 35, 39]. These observations and many other experiments strongly support the hypothesis that contact sites between the ER/MAM and mitochondria are required for mediating PS import from the ER into mitochondria and for providing PE for the normal functioning of mitochondria [reviewed in [5, 40]]. In addition, PS-derived PE is rapidly exported from mitochondria, perhaps via MAM, to other cellular organelles including the plasma membrane. In contrast, the mechanism by which phosphatidylcholine (the most abundant membrane phospholipid in mammalian cells) is imported into mitochondria from its sites of synthesis in the ER has not yet been elucidated. However, in rodent liver and hepatocytes the enzyme PE *N*-methyltransferase (synthesizes phosphatidylcholine from PE) is highly enriched in MAM relative to the bulk of the ER [41]. Thus, it is possible that in hepatocytes, some of the phosphatidylcholine in mitochondria is made in MAM and imported directly from MAM into mitochondria. These experiments on PS transport from the ER to mitochondria were the first demonstration of a specific functional role for membrane contact sites.

In addition to MAM being the principal site of PS biosynthesis [36], a number of other lipid biosynthetic enzyme activities are several-fold enriched in MAM, compared to the bulk of the ER. These activities include acyl-CoA cholesterol acyltransferase (esterifies cholesterol to cholesteryl esters) and diacylglycerol acyltransferase (synthesizes triacylglycerols) [30]. The enrichment of these enzymatic activities in MAM, in combination with electron microscopic visualization of lipid droplets in close proximity to both the ER and mitochondria [11, 42, 43] suggest that MAM might supply lipids for the formation of lipid droplets, as well as for the assembly of very low density lipoproteins in hepatocytes. Indeed, a specific population of mitochondria in brown adipose tissue can provide ATP for stimulation of triacylglycerol synthesis and thereby support the expansion of lipid droplets [43]. Moreover, the lumen of the MAM contains apolipoprotein B, a key protein component of very low density lipoproteins [30]. However, the idea that membrane contact sites such as MAM participate in the biogenesis and secretion of very low density lipoproteins needs to be supported by additional data.

Apart from a role for MAM in PS movement from the ER to mitochondria, most of the pathways that mediate phospholipid and sterol trafficking among organelles have not yet been defined. For example, the mechanisms by which the bulk of phospholipids and cholesterol move from their sites of synthesis in the ER to the plasma membrane have not been elucidated although contact sites between the ER and plasma membrane have frequently been observed. Thus, until recently, little experimental evidence supported a role for membrane contact sites in the inter-organelle trafficking of sterols. The large majority of cellular cholesterol resides in the plasma membrane [44] whereas cholesterol is synthesized in the ER which is also the site at which cellular cholesterol homeostasis is regulated [45]. Consequently, mechanisms must exist for the transfer of cholesterol from the ER to the plasma membrane. In mammalian cells, cholesterol can also be acquired from exogenously-supplied plasma lipoproteins such as high density lipoproteins (HDL) and low density lipoproteins (LDL). The pool of cholesterol derived from exogenously-delivered HDL is internalized at the plasma membrane and transported to the ER. Thus, the transport of cholesterol from the plasma membrane to the ER is also an active process. Some progress has recently been made in understanding the mechanisms underlying this pathway of intracellular cholesterol transport. A family of cholesterol-binding proteins, the Aster proteins, was identified and shown to be recruited to plasma membrane-ER contact sites in response to the accumulation of HDL-derived cholesterol in the plasma membrane [46]. Genetic elimination of the Aster B protein in mice impaired the transport of HDL-derived cholesterol from the plasma membrane to the ER, suggesting a role for Aster B in the trafficking of this pool of cholesterol. Moreover, in mice lacking Aster B, the cholesterol content of the adrenal cortex was reduced and adrenal steroidogenesis was defective [46]. These observations strongly support the idea that Aster B and plasma membrane-ER contact sites mediate the non-vesicular movement of HDL-derived cholesterol from the plasma membrane to the ER. Another interesting feature of the Aster proteins is that they contain a binding site for the anionic phospholipid PS. Moreover, this phospholipid enables the recruitment of Aster B protein to plasma membrane-ER contact sites in response to cholesterol accumulation in the plasma membrane [46].

An additional role for PS and plasma membrane-ER contact sites in inter-organelle cholesterol trafficking in human cells has recently been reported [47]. In these studies, the mechanism by which LDL-derived cholesterol is delivered from the plasma membrane to the ER was investigated in response to cholesterol accumulation in the plasma membrane. Cholesterol is taken up by cells from exogenously-supplied LDL via LDL receptors which are located in the plasma membrane [48]. The LDL particles are internalized via LDL receptor-mediated endocytosis and are subsequently transported to lysosomes for degradation [45]. Cholesterol derived from the LDL particles is then exported from the lysosomes to the ER, the site at which cellular cholesterol homeostasis is tightly controlled [45, 49]. The new study confirms that LDL-derived cholesterol is normally transported from lysosomes to the ER via an excursion through the plasma membrane [47]. A series of elegant experiments reveals that in the absence of one of the PS synthesizing enzymes, PS synthase-1 (but not PS synthase-2), LDL-derived cholesterol exits lysosomes but subsequently accumulates in the plasma membrane, instead of reaching the ER. Furthermore, the expression of PS synthase-1 (an enzyme highly enriched in MAM), or incubation of the cells with PS liposomes, restored cholesterol transport from the lysosomes via the plasma membrane to the ER. The authors conclude that the trafficking of LDL-derived cholesterol from lysosomes to the ER occurs via the plasma membrane and requires an abundant supply of PS. These studies complement the observations that the Aster B protein clusters at plasma membrane-ER contact sites in the presence of PS and that this process is required for the transport of HDL-cholesterol from the plasma membrane to the ER [46]. Thus, it is likely that cholesterol derived from LDL and HDL is transported from the plasma membrane to the ER via membrane contact sites.

Intriguingly, the enrichment of the ER/MAM with the anionic phospholipid PS also promotes the formation of contact sites between the ER and mitochondria and increases the import of PS from the ER into mitochondria [50]. Consistent with these findings, in mammalian cells the MAM is highly enriched in the PS-synthesizing enzymes, PS synthase-1 and PS synthase-2 [36]. The transport of PS from the ER to endosomes also appears to be stimulated by an increased production of PS in regions of the ER that are in contact with endosomes [51]. These observations raise the possibility that PS might promote the formation and functions of several types of inter-organelle contact sites.

It appears likely, therefore, that the enrichment of specific membrane domains with the anionic phospholipid PS might represent a common feature that promotes the formation and/or function of at least three distinct types of contact sites: i.e. those linking the ER and mitochondria, those linking the ER and plasma membrane, and those linking the ER and lysosomes. Another class of anionic phospholipids, the phosphatidylinositol phosphates, have also been reported to enhance the formation of at least some types of membrane contact sites [52]. Thus, it is possible that the presence of specific anionic phospholipids, such as PS or the phosphoinositides, in specific membrane domains promotes the formation of inter-organelle contact sites.

These thought-provoking studies on intracellular cholesterol trafficking have raised additional questions. Are the contact sites that facilitate the transport of the pool of HDL-cholesterol from the plasma membrane to the ER the same contact sites that mediate the transport of LDL-derived cholesterol to the ER? Does either of these contact sites facilitate the transport of cholesterol in the reverse direction - from the ER to the plasma membrane? Do PS or other anionic phospholipids enhance the formation of other types of membrane contact sites? Hopefully, future experiments will answer these questions.

### Conclusions and future directions

Despite the impressive amount of high-quality information that is now available on the nature and functions of inter-organelle membrane contact sites, many important questions remain concerning their identity, compositions and functions. It is likely that future investigations will uncover and characterize additional examples of pairs of organelles that associate with one another. The most well-studied membrane contact sites are those that link the ER/MAM to mitochondria. However, it is not clear if the mitochondria that associate with MAM at contact sites have properties that are distinct from those of mitochondria that do not associate with MAM. An important question remaining is: what factors *regulate* the formation and dissolution of membrane contact sites? Furthermore, a detailed lipidomic analysis of the lipid composition of all known types of membrane contact sites would greatly increase our understanding of the formation and biological roles of inter-organelle membrane contact sites. Over the past few years there have been great advances in the technologies available for visualization of contact sites in cells by microscopy but, as pointed out in the Perspective, some methodological challenges remain in these types of studies. An issue that must be rigorously considered for subcellular fractionation of organelles and contact sites is the potential for contamination of the organelles by other membranes.

The majority of studies on membrane contact sites have been performed in cultured cells, with only a few studies reported from in vivo experiments [43]. Consequently, a major goal for future research on membrane contact sites is to understand how defects in membrane contact sites contribute to human diseases. Some progress has been made in this direction [summarized in [5, 34]]. For example, it is clear that impaired PS import into mitochondria via MAM causes defects in mitochondrial functions such as mitochondrial fusion [51, 53] and ATP production [33]. In Charcot-Marie Tooth disease, defective mitochondrial fusion is caused by mutations in mitofusin-2 (MFN2), a protein that is abundant in MAM and is required for mitochondrial fusion [53]. Thus, it is not surprising that alterations in MAM-mitochondria contacts sites and levels of mitofusin-2 would be implicated in human disorders that exhibit mitochondrial dysfunction. For example, MAM-mitochondria contact sites appear to be involved, somehow, in the regulation of glucose metabolism since the number of MAM-mitochondria contact sites was significantly reduced during insulin resistance in human muscle and liver [54–57]. In other studies, the number of MAM-mitochondria contact sites in liver was reported to be increased in obesity, leading to organelle dysfunction [58]. Mitofusin-2 is now known to be a PS-binding protein that is required for PS import into mitochondria via MAM [57]. A decreased expression of mitofusin-2, and a reduction in the number of MAM contact sites, were observed in in vivo studies in patients with liver disease (non-alcoholic steatohepatitis), as well as in a mouse model of liver disease [57]. Furthermore, the ablation of mitofusin-2 in mice caused steatohepatitis whereas re-expression of mitofusin-2 in these mice increased the number of MAM-mitochondria contact sites and lessened the disease phenotype in the liver [57].

Alterations in the abundance of MAM-mitochondria contacts have also been implicated in the pathogenesis of several neurodegenerative diseases. For example, defects in MAM appear to play a role in regulating mitochondrial calcium homeostasis and mitochondrial activity in Parkinson’s disease [59, 60]. Moreover, in fibroblasts from mouse models of Alzheimer’s disease, as well as in fibroblasts from Alzheimer disease patients, an increased association between MAM and mitochondria, and an increased conversion of PS to PE, were observed [61, 62]. Furthermore, the link between cholesterol-rich domains of the ER and Alzheimer disease pathogenesis was investigated [63]. The C99 peptide fragment derived from othe Alzheimer precursor protein was found to bind cholesterol and to be located in the MAM. In addition, the C99 peptide acted as a cholesterol-sensing peptide by increasing cholesterol transport from the ER/MAM to the plasma membrane [63]. Clearly, more information is required on these important functions of membrane contact sites in human diseases.

The existence of membrane contact sites in eukaryotic cells is now widely accepted. However, despite many significant advances since the 1950s in our knowledge of the nature of inter-organelle membrane contact sites, our understanding of the composition, function and regulation of formation of these elusive, but ubiquitous, cellular structures is still incomplete. As pointed out by Gia Voeltz in a 2019 *Nature* news feature article, text book coverage of the existence and functions of membrane contact sites needs to be updated.

## Data Availability

All data are published and are available.

## Abbreviations

ER: Endoplasmic reticulum
MAM: Mitochondria-associated membranes
PE: Phosphatidylethanolamine
PS: Phosphatidylserine
HDL: High density lipoproteins
LDL: Low density lipoproteins
